# Value of Cytokine Expression in Early Diagnosis and Prognosis of Tumor Metastasis

**DOI:** 10.1155/2022/8112190

**Published:** 2022-09-16

**Authors:** Tingwei Li, Minling Liu, Huiru Dai, Xueying Li, Jiehao Liao, Zilong Zheng, Yihang Pan, Yuchen Liu, Shuo Fang

**Affiliations:** ^1^Department of Oncology, The Seventh Affiliated Hospital of Sun Yat-sen University, Shenzhen 518107, China; ^2^Clinical Big Data Research Center, Precision Medicine Center, The Seventh Affiliated Hospital of Sun Yat-Sen University, Shenzhen 518107, China

## Abstract

**Objective:**

To investigate the association of the plasma level of cytokines and blood routine indexes with clinical characteristics in patients with cancer.

**Methods:**

We analyzed plasma samples derived from 134 cancer patients. Interleukins (IL) 1*β*, 2, 4, 5, 6, 8, 10, 12p70, 17, IFN-*γ*, IFN-*α*, and TNF-*α*, and blood routine indexes were measured. The associations of the levels of cytokine and blood routine indexes with demographic and clinical characteristics of cancer patients were analyzed. Partial least-squares discriminant analysis was employed to identify cancer metastasis using these plasma cytokine metrics as input. We compared the predictive effectiveness of numeric machine learning algorithms using these indexes and showed a promising model implemented with random forest.

**Results:**

Plasma levels of IL-6 and IL-8 in cancer patients with metastases were higher than those without metastases (*P* < 0.05). Cancer patients without metastases had significantly higher levels of plasma IL-12p70 and percentage of lymphocytes as compared with those with metastases (*P* < 0.05). Our random forest model showed the highest prediction performance (upper quantile AUC, 0.885) among the six machine learning algorithms we evaluated.

**Conclusion:**

Our findings suggest that plasma levels of IL-6, IL-8, and IL-12p70 and the percentage of lymphocytes could predict the recurrence, metastasis, and progression of cancer. Our findings will provide guidance for tumor monitoring and treatment.

## 1. Introduction

Cancer is the second leading cause of death worldwide [1]. The International Agency for Research on Cancer Global Cancer Observatory of the World Health Organization predicts there will be 27.5 million new cancer cases around the world annually by 2040, which is a 67 percent increase compared with current data. Malignant tumors account for considerable health care utilization and spending, which has brought a huge economic burden on the health system. In order to reduce the financial burden and expenditure, we are committed to early detection, early diagnosis, and early treatment. The occurrence and development of tumors are affected by multiple factors, including cytokines [2]. In the tumor-associated inflammatory microenvironment, immune cells interact with cytokines, chemokines, and growth factors to promote or inhibit tumor progression [2]. Cytokines mediate key interactions between immune and nonimmune cells in the tumor microenvironment [3]. Different cytokines play different roles in the occurrence and development of tumors. For instance, IL-6 can promote tumor growth while IL-12 can inhibit tumor growth [3]. However, whether cytokines could help determine tumor metastasis is not clear.

In this study, we analyzed the association of plasma cytokines and blood routine index levels with clinical characteristics in cancer patients. Our research has screened out four important biomarkers. The results of our study will provide important guidance for predicting tumor metastasis.

## 2. Materials and Methods

### 2.1. Study Population

A cohort of 134 patients (52 males and 82 females) with newly diagnosed and histologically confirmed diverse cancer types was recruited between 2020 and 2021 at The Seventh Affiliated Hospital, Sun Yat-sen University. All the patients underwent routine blood tests and their plasma levels of cytokines were examined before treatment. The Ethics Committee of the Seventh Affiliated Hospital of Sun Yat-sen University had approved the study, and informed consent was obtained from all patients prior to their inclusion.

### 2.2. Blood Sample Collection

The blood samples of patients were collected by venipuncture using serum separator tubes prior to preoperative radiotherapy and/or surgery. Samples were centrifuged at 3,000 g for 10 min; the serum was separated into aliquots and immediately stored at −80°C until immunoassay. Repeated freeze-thaw was avoided.

### 2.3. Clinicopathological Features, Blood Metrics, and Serum Cytokines

We collected the demographics of patients such as sex and age. The clinicopathological characteristics including tumor type, differentiation status, tumor node metastasis (TNM) stage, T stage, and N stage were assessed. We used the 8th edition of the American Joint Committee on cancer staging system for TNM staging. Blood routine indexes including the white blood cell number, neutrophil number, lymphocyte number, monocyte number, platelet number, and hemoglobin were measured. Plasma levels of IL-1*β*, IL-2, IL-4, IL-5, IL-6, IL-8, IL-10, IL-12p70, IL-17, TNF-*α*, IFN-*α*, and IFN-*γ* were measured by Luminex suspension array using a specific kit (Merck Millipore, Germany) according to manufacturer's instructions.

### 2.4. Statistical Analyses

**Figure 1:**
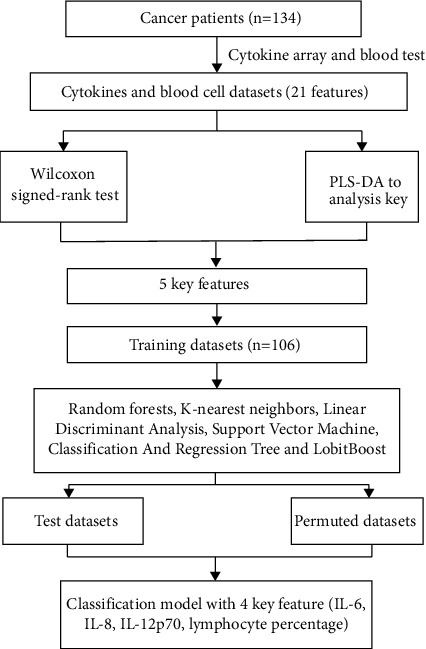
Data analysis flowchart.

All statistical analyses were performed using R Statistical software (v3.6.3). Differential cytokines and blood metrics between patients with and without metastasis were selected according to the Wilcoxon rank sum test, with the cut-off*P* value less than 0.05 and absolute log fold change greater than 0.138. Pearson's correlation between different metrics was performed with the ‘stats' package. All the metrics were scaled to standard deviation (SD) units as to partial least square-discriminant analysis (PLS-DA) and raw data were analyzed with different machine learning methods for prediction model establishment. The PLS-DA in the ‘ROPLS' package was applied to compute the predicted probabilities of metastasis status with serum metrics. Area under receiver operating characteristic curve (AUC) analysis of models for performance measures was done with the ‘ROCR' package. The machine learning methods used in the study include random forests (RF), K-nearest neighbors (KNN), linear discriminant analysis (LDA), support vector machine (SVM), classification and regression tree (CART), and LogitBoost. Each of them was performed with cross validation by using the ‘caret' package. For the cross validation, 4/5 of the samples of each group were used in the training analysis, while the other 1/5 were used for the validation analysis, and iteration for at least 100 times (Figure 1).

## 3. Results and Discussion

### 3.1. Patient Characteristics

As shown in Table 1, this study included 137 cancer patients, 108 patients (80.6%) with metastasis, and 26 (19.4%) without metastasis. There were 7 females (26.9%) and 19 males (73.1%) in the nonmetastatic group, and 45 females (41.7%) and 63 males (58.3%) in the metastatic group. The average age of the nonmetastatic group was 55.69 ± 11.81 years old, and the metastasis group was 58.59 ± 13.03 years old. In the nonmetastatic group, stage I disease was found in 3 (115%) patients, stage II in 6 (23.1%), stage III in 16 (61.5%), and stage IV in 1 (3.8%). It was found that the plasma levels of IL-12p70, lymphocyte number, lymphocyte percentage, and hemoglobin in patients without metastases were significantly higher than those with metastases (*P* < 0.05 for all). Patients with metastases had significantly higher plasma levels of neutrophil number, neutrophilic percentage, platelet number, and white blood cell number than those without metastases (all *P* < 0.05).

### 3.2. Association of Cancer Metastasis with Plasma Cytokines and Blood Routine Indexes

**Figure 2:**
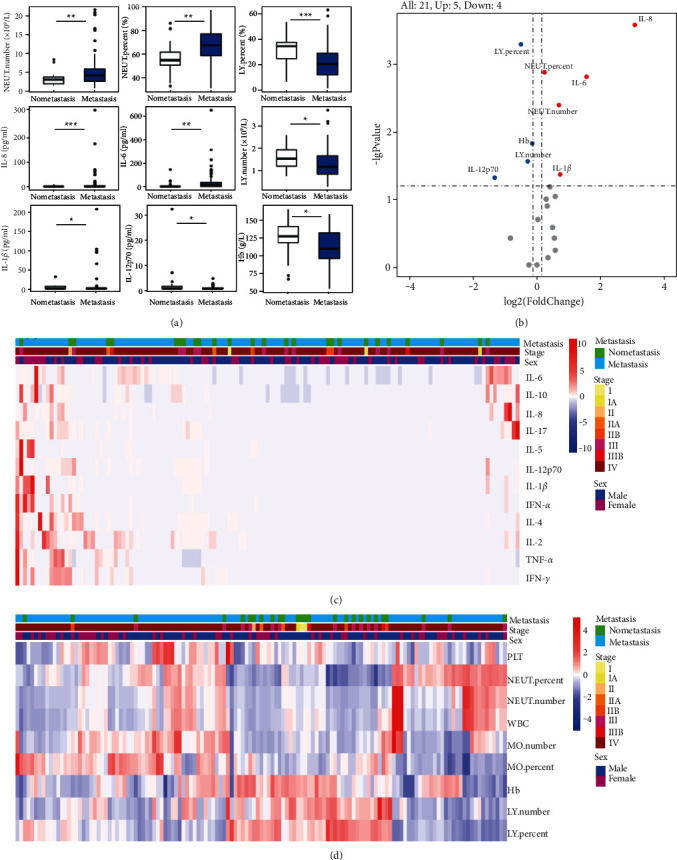
Association of tumor metastasis and plasma cytokines and blood routine indexes. (a) Cytokines and blood routine indexes related to metastasis and nonmetastasis. Wilcoxon test, ^*∗*^*P* < 0.05,^*∗∗*^*P* < 0.01, ^*∗∗∗*^*P* < 0.001; (b) the volcano plot revealed 5 indicators (red points) were upregulated and 4 (blue points) were downregulated in tumor metastasis; (c) the heatmap showed the relationship between cytokines and metastasis; (d) the heatmap showed the relationship between blood routine indexes and metastasis. Abbreviations. Hb, hemoglobin; LY, lymphocyte; MO, monocyte; NEUT, neutrophil; PLT, platelet; WBC, white blood cell.

As shown in Figure 2(a), plasma levels of neutrophil number, neutrophilic percentage, IL-8, IL-6, and IL-1*β* in the metastatic group were significantly higher than those in the nonmetastatic group (all *P* < 0.05). The nonmetastatic group had significantly higher plasma levels of IL-12p70, lymphocyte number, lymphocyte percentage, and hemoglobin than those in the metastatic group (all *P* < 0.05). Among the 21 indicators we observed, 5 of them were upregulated and 4 were downregulated in tumor metastasis (Figure 2(b)). The results were consistent with those in Figure 2(a). A heat map was made to express the relationship between tumor metastasis and plasma cytokines and blood routine indexes. As shown in the heat map (Figures 2(c) and 2(d)), the plasma levels of IL-8, IL-6, and IL-1*β*, neutrophil number, and neutrophilic percentage were higher in the metastatic group and positively correlated with tumor metastasis, while IL-12p70, lymphocyte number, lymphocyte percentage, and hemoglobin were inversely correlated.

### 3.3. Key Indicators to Distinguish Metastatic and Nonmetastatic Tumors

**Figure 3:**
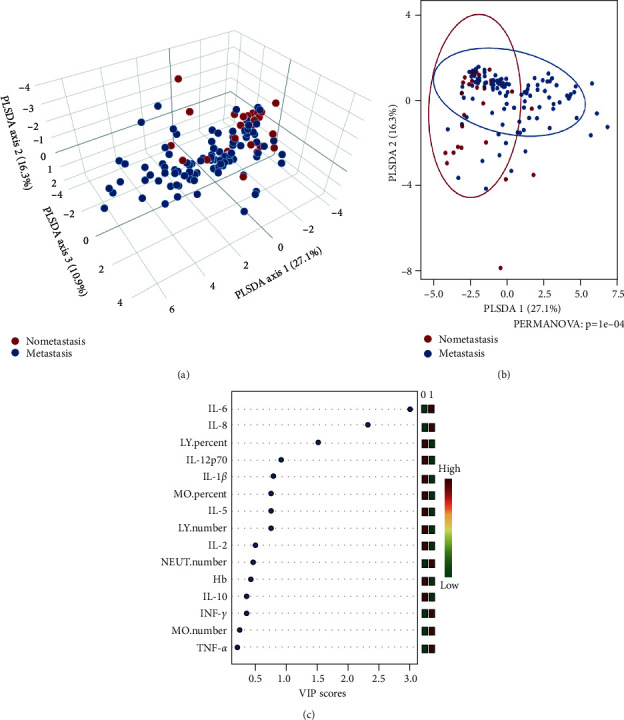
Key indicators to distinguish tumor metastases and nonmetastases. (a and b) PLS-DA analysis showed tumor metastasis and nonmetastasis distinguished by certain indicators (PERMANOVA test, *P*=1*e* − 04); (c) PLS-DA analysis showed the plasma levels of IL-6, IL-8, lymphocyte percentage, and IL-12p70 were the main influencing factors that distinguished metastases and nonmetastases (VIP scores ≥1). Abbreviation. VIP, variable importance in the projection.

As can be seen from Figure 3(a) and Figure 3(b), PLS-DA analysis revealed that the plasma levels of IL-6, IL-8, lymphocyte percentage, and IL-12p70 were the main influencing factors that distinguished tumor metastases and nonmetastases (Figure 3(c), VIP scores ≥1).

### 3.4. Random Forest Is the Best Machine Learning Algorithm for Predicting Tumor Metastasis with 4 Key Indexes Features

**Figure 4:**
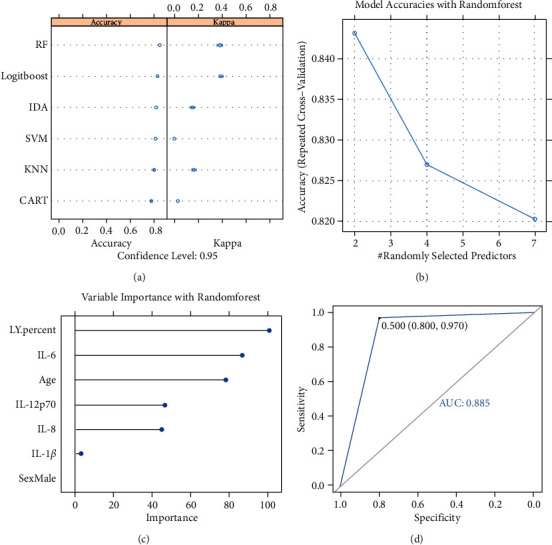
(a) Random forest is the best machine learning algorithm for predicting tumor metastasis. The model with the random forest algorithm showed the highest accuracy (0.84) and its kappa index was 0.38. (b) The relationship between variables and accuracy in the random forest predictor, with the repeated five-fold cross validation. (c) The variable importance rank in the random forest model are lymphocyte percentage, IL-6, IL-12p70, and IL-8. (d) The AUC value of ROC curves is 0.885, which indicated that the random forest model was robust for predicting tumor metastasis. Abbreviation: RF, random forests; LDA, linear discriminant analysis; SVM, support vector machine, KNN, K-nearest neighbours; CART, classification and regression tree; LY, lymphocyte.

To establish a model for predicting tumor metastasis, we compared the performance of six machine learning algorithms with 100 times of repetition by using a 5-fold cross validation strategy using our dataset. As shown in Figure 4(a), all used algorithms exhibited high accuracy (≥0.77) for these permuted test sets. The model with the random forest algorithm showed the highest sensitivity (0.84) and its kappa index was 0.38 reflecting moderate accuracy. The relationship between variables and accuracy in random forest predictor was analyzed (Figure 4(b)). The variable importance rank in the random forest model are lymphocyte percentage, IL-6, IL-12p70, and IL-8 (Figure 4(c)). In order to evaluate the effect of the model on distinguishing tumor metastatic status, we drew the ROC curve (Figure 4(d)) over the permuted data sets, and the results showed an upper quantile AUC value of 0.885, which indicated that the random forest model was well established and the 4 key indexes have predictive value for tumor metastases.

### 3.5. Significant Features in Gender Subgroups

**Figure 5:**
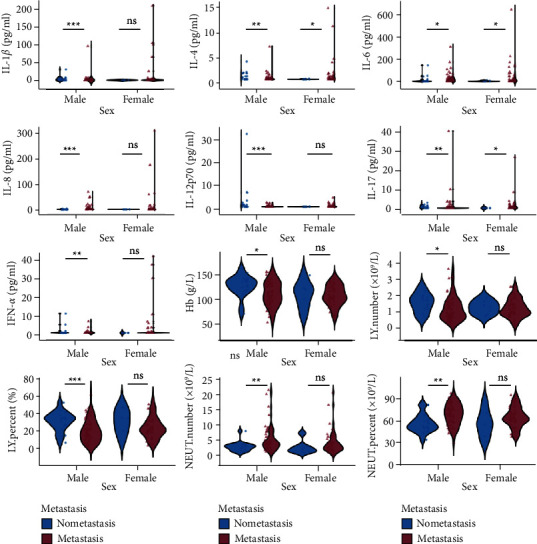
The relationship between tumor metastasis with plasma cytokines and blood routine indexes in gender subgroups. Wilcoxon test, ^*∗*^*P* < 0.05,^*∗∗*^*P* < 0.01, ^*∗∗∗*^*P* < 0.001; ns, no significance.

We divided the metastatic and nonmetastatic patients by gender as subgroups, and found that among the male patients, plasma levels of IL-1*β*, IL-4, IL-6, IL-8, IL-17, neutrophil number, and neutrophilic percentage were higher in the tumor metastatic group. Plasma levels of IL-12p70, lymphocyte number, lymphocyte percentage, and hemoglobin were higher in the nonmetastatic group. In the female patients, IL-4, IL-6, and IL-17 plasma levels were higher in the metastatic group, and there was no statistically significant difference in other indicators between metastatic and nonmetastatic patients (Figure 5).

## 4. Discussion

Malignant tumors have become an increasingly serious global public health problem. It is the second leading cause of death next to cardiovascular diseases and has a serious impact on the health and economy worldwide [1]. The occurrence and development of tumors is a multifactor, multistep, and multimechanism process. Although the underlying mechanisms of carcinogenesis and cancer progression have not yet been fully understood, recent findings suggest that inflammatory changes in the tumor microenvironment play a crucial role in carcinogenesis [4].

Inflammation can increase the risk of cancer by providing bioactive molecules from cells infiltrating the tumor microenvironment, including cytokines, growth factors, proangiogenic factors, and so on [5]. Cytokines mediate key communications between cells in the tumor microenvironment [3]. At present, there are many studies on cytokines, and the role of some cytokines in the initiation and progression of tumors is known. For instance, IL-6 is a proinflammatory cytokine with a typical protumorigenic effect. It plays a vital role in promoting tumor cell proliferation and inhibition of apoptosis, and has been proposed as a predictor of malignancy [3, 6]. IL-12 demonstrates striking immune activation and antitumor effects [3]. However, there are few studies on what indicators are more helpful in determining tumor metastasis.

In this study, we analyzed the associations of interleukins (IL) 1*β*, 2, 4, 5, 6, 8, 10, 12p70, 17, IFN-*γ*, TNF-*α*, and IFN-*α* and blood routine indexes with the demographic and clinical characteristics of 134 cancer patients. Through various statistical analyses, we found that plasma levels of cytokines and blood routine indexes had a close association with demographic and clinicopathological features of cancer patients.

All plasma levels of neutrophil number, neutrophilic percentage, IL-1*β*, IL-8, and IL-6 in cancer patients with metastases were significantly higher than those without metastases (*P* < 0.05 for all). The nonmetastatic group had significantly higher plasma levels of lymphocyte number, lymphocyte percentage, hemoglobin, and IL-12p70 than those in the metastatic group (*P* < 0.05 for all). Further analysis revealed that the plasma levels of IL-6, IL-8, lymphocyte percentage, and IL-12p70 were the main influencing indexes that distinguished metastases and nonmetastases. The ROC curve proved that the model was well established and indicated that these four indicators can well distinguish between tumor metastasis and nonmetastasis.

Inflammation is a hallmark of cancer development [5]. Neutrophils are the major components of systemic inflammatory response. They promote carcinogenesis and cancer progression by regulating extracellular matrix and inflammation in the tumor microenvironment [7]. Nozawa et al. reported that neutrophils could promote tumor angiogenesis [8]. De Larco et al. showed that neutrophils could enhance the spread of tumor cells [9], and neutrophils were shown to promote the transplantation and metastasis of tumor cells in distant organs [10].

IL-1*β* was demonstrated to promote inflammation-induced carcinogenesis and contributes to tumor aggressiveness [3, 11]. IL-6 is a typical proinflammatory cytokine that promotes tumor growth. It has been shown to amplify inflammation and promote inflammation-induced carcinogenesis [12–14]. Tumor-derived IL-8 can alter immune-invasive components in the tumor microenvironment and induce angiogenesis. It facilitates oncogenic signaling and prometastatic features such as invasion and resistance to chemotherapy [15, 16]. Lymphocytes play a vital role in producing cytokines, inhibiting the proliferation of cancer cells and inducing cytotoxic cell death [17]. It has a powerful antitumor immune function and can inhibit the progression of many kinds of tumors [18]. It was reported that an elevated number of lymphocytes is associated with favorable outcomes in some tumors [19]. Lymphocytes can inhibit tumor proliferation and metastasis [20]. Additionally, it has been speculated that anemia may promote distant metastasis by increasing tumor hypoxia and increasing tumor cell resistance to treatment. [21–23]. IL-12p70 is produced by dendritic cells. The higher the level of IL-12p70, the stronger the ability of dendritic cells to stimulate T cells, the lower the number of inhibitory immune cells, and the stronger the ability to produce antigen-specific cytotoxic T lymphocytes, which can inhibit tumor progression and metastasis [24].

Previous studies have shown that neutrophils, IL-1*β*, IL-8, and IL-6 were upregulated and lymphocyte, hemoglobin, and IL-12p70 were downregulated in tumor metastasis, which is consistent with our findings. In clinical practice, more than 12 cytokines are often evaluated, which increases the economic burden of patients and national medical insurance expenditure. In our study, four key indicators were found to better predict tumor metastasis, namely IL-6, IL-8, lymphocyte percentage, and IL-12p70. This provides important guidance for the detection of cytokines in clinical practice. According to the results of our study, in clinical practice, if the levels of IL-6 and IL-8 were increased while the levels of IL-12p70 and lymphocyte percentage were decreased, the possibility of tumor metastasis would be considered high, which is not only conducive to tumor staging and the guidance of treatment but also can be used for monitoring tumor recurrence and metastasis. Based on the results of this study, patients can spend less money to obtain the maximum clinical value in clinical work.

In the subgroup analysis, we found that IL-4 and IL-17 in male patients were also meaningful indicators to distinguish metastatic from nonmetastatic tumors, while IL-4, IL-6, and IL-17 in female patients were meaningful indicators. IL-4 promotes TH2-type inflammation and TH9 cell polarization, which promotes cancer growth. IL-17 causes large numbers of immunosuppressive granulocytes to cluster together [25], which has been shown to promote lung metastasis in a murine breast cancer model [26]. The results of our subgroup analysis are consistent with the conclusions of existing studies. Subgroup analysis showed that our results are more applicable in male patients. IL-4 and IL-17 are also associated with tumor metastasis.

Our study has some limitations. First, this is a retrospective study, and it is difficult to avoid the influence of bias. Second, although the levels of a range of plasma cytokines and blood routine indexes were analyzed, some other important cytokines that may be involved in the inflammatory response may have been overlooked. Third, the study did not take into account some lifestyle-related factors, such as exercise, that might influence cytokine levels. Finally, this is a single-center study and its generality to other populations may be limited. The results of our study need to be verified by large-scale prospective studies.

## 5. Conclusions

Our study found that the levels of plasma cytokines in cancer patients were closely related to tumor metastasis. IL-6, IL-8, and IL-12p70 and lymphocyte percentage were the key indicators to distinguish between metastatic and nonmetastatic tumors. The results of this study will provide guidance for tumor monitoring and clinical decision-making.

## Figures and Tables

**Table 1:** Baseline characteristics of the study cohort according to metastasis.

Clinical characteristics	Nonmetastasis (*n* = 26)	Metastasis (*n* = 108)	*P*
Age (mean (SD))	55.69 (11.81)	58.59 (13.03)	0.302
Gender			
Female	7 (26.9%)	45 (41.7%)	0.246
Male	19 (73.1%)	63 (58.3%)	
Stage			
I	3 (11.5%)	0 (0.0)	<0.001
II	6 (23.1%)	0 (0.0)	
III	16 (61.5%)	0 (0.0)	
IV	1 (3.8%)	108 (100.0%)	
IL-1*β* (mean (SD))	4.66 (6.23)	7.73 (26.33)	0.557
IL-2 (mean (SD))	0.99 (0.91)	1.24 (2.23)	0.571
IL-4 (mean (SD))	1.36 (0.81)	1.38 (1.84)	0.950
IL-5 (mean (SD))	5.16 (15.78)	2.87 (6.34)	0.242
IL-6 (mean (SD))	12.51 (29.80)	36.54 (77.84)	0.125
IL-8 (mean (SD))	1.30 (0.88)	10.84 (35.80)	0.178
IL-10 (mean (SD))	2.71 (3.29)	2.28 (2.85)	0.500
IL-12p70 (mean (SD))	2.97 (6.23)	1.18 (0.60)	0.004^*∗*^
IL-17 (mean (SD))	1.50 (0.70)	2.23 (4.70)	0.435
TNF-*α* (mean (SD))	2.09 (1.08)	2.95 (4.27)	0.311
IFN-*γ* (mean (SD))	4.45 (8.05)	6.65 (16.18)	0.502
IFN-*α* (mean (SD))	1.64 (2.18)	2.39 (6.10)	0.542
Hb (mean (SD))	124.23 (24.40)	111.72 (23.14)	0.016^*∗*^
LY number (mean (SD))	1.54 (0.55)	1.27 (0.65)	0.049^*∗*^
LY% (mean (SD))	32.05 (11.60)	22.49 (12.67)	0.001^*∗*^
MO number (mean (SD))	0.40 (0.18)	0.49 (0.26)	0.108
MO% (mean (SD))	7.85 (2.64)	7.87 (3.19)	0.968
NEUT number (mean (SD))	3.04 (1.66)	4.89 (3.91)	0.020^*∗*^
NEUT% (mean (SD))	56.46 (13.56)	66.90 (14.18)	0.001^*∗*^
PLT number (mean (SD))	183.50 (63.44)	229.33 (111.59)	0.046^*∗*^
WBC number (mean (SD))	5.17 (1.72)	6.82 (4.04)	0.045^*∗*^

Analyzed by the Wilcoxon test, ^*∗*^*P* < 0.05. Abbreviations. Hb, hemoglobin; LY, lymphocyte; MO, monocyte; NEUT, neutrophil; PLT, platelet; WBC, white blood cell.

## Data Availability

The data analyzed during the present study can be available from the corresponding author upon request..
